# Ten failings in global neglected tropical diseases control

**DOI:** 10.1371/journal.pntd.0005896

**Published:** 2017-12-21

**Authors:** Peter J. Hotez

**Affiliations:** 1 Texas Children’s Center for Vaccine Development, National School of Tropical Medicine, Baylor College of Medicine, Houston, Texas, United States of America; 2 James A Baker III Institute for Public Policy, Rice University, Houston, Texas, United States of America; 3 Scowcroft Institute for International Affairs, The Bush School of Government and Public Service, College Station, Texas, United States of America; New York Blood Center, UNITED STATES

Over the course of the last decade, the global community has made tremendous progress towards neglected tropical disease (NTD) control or even elimination, especially for some of the 20 conditions now recognized by the World Health Organization (WHO) [1]. However, there remain important and substantive gaps in our achievements. Some of these gaps are glaring and obvious, and the fact that they continue to be ignored by global leaders and policymakers approaches a moral failing or outrage. Listed here are 10 of what I consider to be our greatest missed opportunities, including some that I previously highlighted as priorities for the new WHO Director-General, Dr. Tedros [2].

The first group of failings is linked to the geopolitics of the NTDs and is listed below.

1 NTDs of regional importance. There are several NTDs that have enormous regional importance in the areas where they occur. But because these diseases are not widely or globally distributed, they do not rise to the top in terms of disease burden estimates and are largely ignored by the global community. Examples include Buruli ulcer in West Africa, loiasis in Central Africa, and podoconiosis (nonfilarial elephantiasis) in East Africa. New World regional diseases include paracoccidioidomycosis, mucocutaneous leishmaniasis, and Oroya fever (Carrion’s disease). However, the example of dracunculiasis and efforts towards its eradication show that these diseases do not necessarily have to be ignored.
2 Political destabilization in the “Old World”: Leishmaniasis and cholera arising from the killing fields. Next to poverty, conflict may be the biggest social determinant of NTDs. Both cutaneous and visceral leishmaniasis outbreaks are now arising from horrific battles and collapsed health system infrastructures in Syria, Iraq, Afghanistan, Sudan, and South Sudan [2–4], while cholera is causing a dramatic and lethal epidemic in Yemen. Cutaneous leishmaniasis has now reached hyperendemic proportions in current and former ISIS occupation zones, and through forced human emigrations, this NTD may spill over into Lebanon, Turkey, Jordan, and elsewhere [3]. Visceral leishmaniasis is killing an unknown number of people in war-torn Sudan and South Sudan [4]. During the 1980s and 1990s, visceral leishmaniasis killed tens of thousands of people from the conflicts happening in some of the same locations in East Africa. It’s happening again.
3 Political destabilization in the “New World”: Venezuela. As Venezuela’s health system slowly collapses, we have seen the resurgence or reemergence of malaria and NTDs such as dengue and other arthropod-borne virus (arbovirus) infections, Chagas disease, and schistosomiasis, just to name a few [5]. Even though Venezuela’s reemerging NTDs now threaten to spread to neighboring Latin American countries or even reverse global goals in the region, the leaders of the Organization of American States (OAS) have so far not seriously worked to tackle this emerging health crisis.
4 Climate change and its impact on vector-borne and zoonotic NTDs. We’ve seen the rapid introduction or reemergence and then spread of dengue, chikungunya, yellow fever, and Zika virus infection in the Western Hemisphere—together with the appearance of mosquito-transmitted and snail-borne diseases in parts of Europe, including arbovirus infections and schistosomiasis—and also an uptick of diseases in Central Asia and the Middle East [2, 6]. Former Vice President Al Gore and others have pointed out that climate change can work in concert with poverty, war, and population movements to produce its detrimental effects, and in this case, the combination appears to promote the increase and spread of NTDs [2, 6]. There is a lot of global interest in climate change, but we could do more to redirect some of this energy towards interventions in the areas of greatest vulnerability.
5 Blue marble health: NTDs in “wealthy” nations. “Blue marble health” refers to new and somewhat paradoxical findings that the poor living in wealthy group of 20 (G20) nations—and also Nigeria (richer than the bottom three or four G20 nations)—account for a majority of the world’s disease burden for the poverty-related neglected diseases and NTDs [2, 7, 8]. These numbers include millions of Americans living in the United States with an NTD and a significant but mostly hidden level of poverty and disease in Europe and Australia [7, 8]. Thus far, it’s not been possible to get this concept on the agendas of G20 summits. But we urgently need a commitment from the G20 leaders to provide treatments for their own vulnerable populations and to commit to research and development (R&D) investments [9]. The bottom line is that the G20 leaders are missing a chance to simultaneously eliminate disease and improve their own economies.

The second group is more linked to closing coverage gaps and providing universal access and is listed below.

6 Female genital schistosomiasis. By now almost everyone connected to global health NTDs should know that female genital schistosomiasis (FGS) is one of the most common gynecologic conditions of women who live in poverty in Africa [10]. They should know that FGS has emerged as one of Africa’s most important cofactors in its AIDS epidemic. However, the leaders of the global HIV/AIDS communities continue to ignore these findings and act as if they simply want this problem to go away and not trouble them any further.
7 Patient access to Chagas disease essential medicines. Chagas disease affects millions of impoverished people in the Americas. Today, most of the cases of Chagas disease occur in Latin America’s three large economies—Argentina, Brazil, and Mexico—and yet more than 90 percent of people with *Trypanosoma cruzi* infection do not have access to treatment [2, 11]. They include tens of thousands of *T*. *cruzi*–infected pregnant women that may pass their infection vertically to their unborn fetus. Chagas disease ranks among the most glaring health disparities in the Americas. The disease is also globalizing to Southern Europe and elsewhere. So far, well-intentioned efforts to raise the awareness of this disease have made little impact [12]. When it comes to Chagas disease, the OAS and the Inter-American Development Bank are both conspicuous by their absence.
8 Closing the gap on mass drug administration (preventive chemotherapy)—And then there’s yaws and scabies. We’re making good but not great progress on providing NTD essential medicines used for mass treatment of the three soil-transmitted helminth infections, lymphatic filariasis, and onchocerciasis. WHO now finds that we’re covering less than two-thirds of the global population requiring treatment for these diseases, but we may not be doing as well for trachoma and schistosomiasis [2, 13]. Also, the major mass drug administration and preventive chemotherapy partners and donors are still mostly silent about extending these programs to also eliminate yaws (azithromycin) and scabies (ivermectin) [2, 14]. These two cutaneous neglected diseases are not exactly “low-hanging fruit,” but we can certainly accelerate progress towards their elimination.
9 R&D “one shot on goal.” I’m not quite sure how this happened. For diseases such as HIV/AIDS, tuberculosis (TB), and malaria, it’s understood that we will need multiple new approaches to control and eliminate these diseases. It’s also accepted that for AIDS, TB, and malaria, we will need to pursue (and fund) several avenues of R&D simultaneously, including new drugs, diagnostics, vaccines, and—for malaria—vector control approaches [15]. But for the NTDs, it seems we only get one shot on goal. If a mass drug administration approach is being funded, we can’t fund a vaccine, or vice versa [15]. There is a terrible and unacceptable double standard for our diseases. History teaches us that we will not eliminate an NTD through only one-dimensional strategies.
10 Leprosy elimination. The community of leprosy scientists and public health experts has incredible passion and commitment. The good news is that in 2016 WHO launched a new leprosy strategy, which hopefully will redouble global efforts for control and elimination.

Certainly, these 10 items are not the only NTD challenges for our global leaders and political institutions. At *PLOS Neglected Tropical Diseases*, we look forward to hearing from you regarding things I might have missed or ignored.
